# Plasticity in invertebrate sensory systems

**DOI:** 10.3389/fphys.2013.00226

**Published:** 2013-08-23

**Authors:** Elzbieta M. Pyza

**Affiliations:** Department of Cell Biology and Imaging, Institute of Zoology, Jagiellonian UniversityKrakow, Poland

**Keywords:** insects, *C. elegans*, lesion-induced plasticity, experience-induced plasticity, circadian plasticity

The Research Topic presented in this issue of Frontiers in Invertebrate Physiology is on Plasticity in Invertebrate Sensory Systems and comprises a total of eight articles. These cover various aspects of sensory plasticity observed not only at the level of neurons but also in behavioral adaptations that result from plastic changes in the nervous system. Neuronal plasticity has been reported in both vertebrate and invertebrate nervous systems and has mostly been documented during development. The phenomenon of plasticity also occurs in the adult nervous system, however, after injury—lesion-induced plasticity—and also after stimulation—experience-induced plasticity. Moreover, plasticity can be a process reflecting rhythmic changes in the environment, either during the day and night or in the seasons throughout the year. This type of plasticity is driven by rhythmic changes in the external environment—daily plasticity—and/or it may be generated endogenously, by circadian clocks—circadian plasticity. In invertebrates, neuronal plasticity has been reported mainly in molluscs and insects as various responses involving axon sprouting and synapse formation.

The term plasticity has been used in neuroscience for over a century and many scientists have applied this term to any change in the brain. Nowadays physiological changes at synapses after stimulation, originally in the form of long-term potentiation (LTP), and modifications in synaptic transmission as a result of learning, are regarded as fundamental plastic processes in the nervous system. However, a wide range of evidence has accumulated so far that the term plasticity can also be used to give an account of temporary or permanent structural changes of synapses, neurons and glial cells in response to internal and external stimuli.

The first examples of these phenomena that have been observed occur during development and after emergence, during the so-called critical period of increased sensitivity, and were reported as changes in the brain's final wiring that occur in response to early sensory experience. Later, changes in brain wiring were reported to occur not only during development and in early life but also in the mature brain. Now it is commonly accepted that the mature brain is plastic and that the extent of neuroplasticity is one of the brain's most amazing features. It allows an organism to adapt to new environments and to learn even until the end of life. Plasticity of the brain does, however, decrease with age, strongest changes occurring in young animals, especially during a period of enhanced activity dependence, the critical period. Although plasticity may occur in various regions of the nervous system the most striking changes have been observed in sensory systems and in the centers for learning and memory in the brain.

As shown in the articles published within this Topic Issue the nematode worm *Caenorhabditis elegans* and various insect species are good models to study neuroplasticity and its mechanisms. Despite their often being considered hard-wired, the nervous systems of invertebrates are in fact plastic, just as in vertebrates, both during development and in the adult. After injury, as reported in the article by Pfister et al. (2012) neurite outgrowth occurs. For example deafferentated neurons in the auditory system of orthopteran insects undergo dendritic and axonal growth. This leads to a gain of function, but the most surprising result observed by the authors is a dimorphic regeneration in response to this type of injury. Lesion-dependent neuroplasticity of the peripheral auditory nerve is also indicated in another article, by Lakes-Harlan (2013) who reports that the lesioned nerve regrows and forms new synaptic contacts. In addition there are also changes in the central nervous system in which sprouting of axon collaterals occurs. The article by Pflüger and Wolf (2013) also gives an example of plasticity in Orthoptera. In locusts, these authors observe activity-dependent plasticity in another sensory system, in wind-sensitive hair receptors of the sensorimotor system.

Several articles within the Topic Issue focus on experience-dependent plasticity, and various forms of learning during development and also in adults. The article by Bozorgmehr et al. (2013) details the influence of experience on habituation, the simplest example of learning in *C. elegans*, while the article by Arenas et al. (2013) reports plasticity induced by social experience in the honeybee. In both cases, *C. elegans* expressing a simple behavior and the more complex individual and social behaviors of the honeybee, structural and functional changes occur in various sensory systems and these affect learning and behavior. In turn Dylla et al. (2013) report a form of associative learning called trace conditioning.

Finally two articles provide examples of daily and circadian plasticity in insects. In flies, neurons and glial cells undergo size changes during the day and night and because this cyclical plasticity is also maintained under conditions of constant darkness it must be endogenous, generated by a circadian clock (Górska-Andrzejak, 2013). Moreover this author gives convincing examples that glial cells are important for plasticity of the neurons they surround. In turn clock neurons, which generate circadian rhythms observed in behavior and other processes, including cyclical structural changes in neurons and glial cells of the visual system in flies, are under pressure of environmental stimuli (Shiga, 2013). Their morphological changes depend on photoperiod. In long days they have longer commissural fibers. The articles published with this Topic Issue show that many factors affect the structure and physiology of neurons in invertebrates, no less than in the brains of vertebrate species. Thus, the nervous systems of invertebrates prove themselves to be not only plastic in response to injuries, so as to re-establish damaged connections and functions, but are also remodeled in response to external and internal signals.
